# Exploring the Influence of Learning Motivation and Socioeconomic Status on College Students’ Learning Outcomes Using Self-Determination Theory

**DOI:** 10.3389/fpsyg.2020.00849

**Published:** 2020-07-03

**Authors:** Hao Li, Michael Yao-Ping Peng, Mingyue Yang, Chun-Chun Chen

**Affiliations:** ^1^Business School, Beijing Normal University, Beijing, China; ^2^School of Economics and Management, Foshan University, Foshan, China; ^3^School of Digital Economics, Guilin University of Electronic Technology, Guilin, China; ^4^National Academy of Economic Strategy, Chinese Academy of Social Sciences, Beijing, China; ^5^School of Management, Beijing Union University, Beijing, China

**Keywords:** self-determination theory, higher education, economically disadvantaged students, student learning outcomes, learning modes

## Abstract

Higher education, which has the function of cultivating human capital, has already become a key focus of developed countries around the world. From ministries of education to higher education institutions, many bodies are dedicated to enhancing student learning outcomes. However, social and educational problems derived from disadvantaged groups have long been hindering the development of individuals and the whole country. This study examines the learning motivations of economically disadvantaged versus non-disadvantaged college students and evaluates the relationship between learning modes and learning outcomes from a self-determination theory (SDT) perspective. In this study, 817 valid questionnaires were collected to compare the two sample groups in terms of learning path. The results show that non-economically disadvantaged students have superior outcomes compared to disadvantaged students in terms of role identity, academic identity, explorative learning, exploitative learning, and cognitive and non-cognitive gains. In regard to path analysis, economically disadvantaged students are significantly superior to non-disadvantaged students in the face of positive influence of academic identity on different learning modes and positive influence of explorative learning on cognitive and non-cognitive gains. Finally, based on the conclusions, this study proposes some suggestions specific to theoretical mode for future study.

## Introduction

Higher education plays an important role in talent cultivation and is also the foundation for national economic development (Choi and Rhee, 2014). However, reform of higher education policies can often give rise to problems, including low-quality teaching and insufficient competitiveness (Shin and Harman, 2009; Marginson, 2011; Jang et al., 2016; Al-Jubari et al., 2019), even when providing education opportunities to more people. These problems have become a central concern for the development of higher education institutions (Trenshaw et al., 2016). Although students in developed countries enjoy fair and reasonable rights to education, and disadvantaged students are provided with special educational support, relevant psychological factors of economically disadvantaged students are neglected. Specifically, deficiencies in congenital education resources generate obvious learning dilemmas, which can reduce learning motivation (Jang et al., 2016). Furthermore, the family dilemma can give rise to disappointment and pessimism among students, which further restricts their academic performance indirectly.

Donnelly (1987) pointed out that disadvantaged, at-risk students mostly come from families with low socioeconomic status. In such families, parents have lower social expectations for their children, and children who lack successful learning experiences are often vulnerable to certain psychological traits (Petersen et al., 2009), such as low self-esteem and low confidence (Guay et al., 2008). Recent research on higher education found that student learning outcomes can be significantly improved by improving the quality of teaching, reforming course designs, and optimizing resources and equipment (e.g., Pike et al., 2011, 2012; Maringe and Sing, 2014; Aidinopoulou and Sampson, 2017; Sergis et al., 2018). However, few studies have explored the psychological traits or learning motivations of economically disadvantaged students (Chen et al., 2009; Hummel and Randler, 2012). Cole et al. (2004) considered that learning motivation, as an important predictor of learning effectiveness and high learning outcomes, triggers the abovementioned psychological factors in economically disadvantaged students and influences their academic performance (Jang et al., 2016; Aidinopoulou and Sampson, 2017; Sergis et al., 2018). For example, excessive learning pressure generates feelings of discouragement and reduces learning motivation, makes time management difficult, etc. Petersen et al. (2009) pointed out that students with lower socioeconomic status may be at higher risk of dropping out. Hence, there is an urgent need for school teachers to discuss the psychological factors that influence the academic performance of economically disadvantaged students, find ways to increase these students’ learning motivation, and cultivate learning interest and a drive for continuous learning.

Although scholars have assigned various definitions to motivation, all of them have emphasized that individual behavior arises from the internal thought process of specific physiological or psychological goals (Deci and Ryan, 2002; Hummel and Randler, 2012; Jang et al., 2016; Shogren et al., 2018; Al-Jubari et al., 2019) in the form of “stimulus–response–enhancement” (Trenshaw et al., 2016). Therefore, learning motivation can be regarded as a kind of learning view of the scholars and can generate learning needs accordingly (Petersen et al., 2009). In recent years, Western researchers have probed the learning psychology and behavior of individuals from the perspective of achievement motivation (Deci and Ryan, 2000; Wigfield and Eccles, 2000; Jang et al., 2016; Aidinopoulou and Sampson, 2017; Sergis et al., 2018); cross-culture studies have also been emerging. Studies have shown that the psychological process and achievements pursued by non-Western students are significantly different (Heine et al., 2001; Chen et al., 2009; Chang et al., 2011).

Motivation plays a key role in the learning process. Related previous studies on motivation have an important position in the field of educational psychology. Most studies of motivation generally focus on self-determination theory (SDT) (Deci and Ryan, 2000; Zepke and Leach, 2010; Shogren et al., 2018; Al-Jubari et al., 2019) and goal orientation theory (Deci and Ryan, 2000; Wolters, 2004). The goal orientation theory emphasizes the impact of the goal structure of the learning environment on the learning process (Deci and Ryan, 2000), while SDT puts emphasis on the impact of the learning environment, psychological needs, and self-determined motivation on the learning process. The psychological needs perceived by learners will internalize self-determined motivations into intrinsic motivations (Deci and Ryan, 1985; Jang et al., 2016; Trenshaw et al., 2016). Thus, this study aims to discuss the role of students’ psychological needs and learning motivation in the learning process and build a research framework on the basis of SDT. Compared to the needs-hierarchy theory, or cognitive-oriented approach, SDT is more able to completely explain the psychology and behavioral modes of non-Western students, especially Asian students (Heine et al., 2001; Brown and Kobayashi, 2002).

With regard to the learning motivation of Asian students, Chen et al. (2009) proposed a “Conceptual Framework for Achievement Goals.” In their discussion of achievement motivation among Taiwanese undergraduates (with an average age of 20), the authors found that, in addition to thinking about spontaneous interest and identity, students considered social desirability, their corresponding sense of obligation and role identity, etc. In line with Cole et al. (2004), the authors of the present study suggest that students should not only complete their academic studies but also consider their individual responsibilities and social needs during their time at university (Shogren et al., 2018). Therefore, academic identity (Kogan, 2000; Nasir et al., 2009; Winter, 2009) and role identity (White et al., 2008) are proposed in this study, and a self-report scale is adopted to verify the consciousness and circumstances of economically disadvantaged, versus non-economically disadvantaged, students in relation to their learning motivation, as well as their relevance with the outcome variable.

Other important factors that influence the learning of undergraduates include the experience and knowledge provided during the learning process, and especially positive attitudes generated toward active participation and learning engagement (Pike et al., 2012; Jang et al., 2016). Among these, learning engagement is key for students to truly internalize the experience and use it to augment their own knowledge and abilities (Duff et al., 2004; Campbell and Cabrera, 2014; Oleson and Hora, 2014). Studies have confirmed that the learning method of students sufficiently reflects the teaching essence and connotation of teachers (Barrie, 2007; Oleson and Hora, 2014; Al-Jubari et al., 2019). In addition, research on learning outcomes has considered learning theories in relation to lesson procedures and content (Jang et al., 2016; Aidinopoulou and Sampson, 2017; Sergis et al., 2018). The learning mode has been emphasized in relation to the content, and theoretically probed or practically applied teaching has been used to inspect the learning results of students. Therefore, in this study, the learning mode is divided into explorative learning and exploitative learning using the dual-classification method, and the learning outcomes of economically disadvantaged versus non-economically disadvantaged students are analyzed by considering different learning methods in the intact research framework (Shogren et al., 2018).

According to the above explanations, this study intends to propose relevant research contributions based on the following theoretical gaps: (1) applying SDT to explore economically disadvantaged students’ learning motivation in higher education; (2) exploring the learning modes from the perspective of students to cultivate and establish students’ learning outcomes, and verify the relevance between the two; (3) adding the concept of socioeconomic status to research framework to compare the difference with non-economically disadvantaged students.

## Literature Review

### Theoretical Context and Student Learning Outcomes

SDT, as proposed by Deci and Ryan (1985), suggests that an individual’s intrinsic psychological motivation for action is to meet certain interests, while the action process based on this intrinsic motivation aims at the action (Jang et al., 2016; Trenshaw et al., 2016). Individuals are assumed to have three basic needs: autonomy, competency, and social relatedness. Individuals’ actions aimed at avoiding the restriction of external rewards and punishments, acting in a socially desirable way, etc., derive from their extrinsic motivation (Deci and Ryan, 2000; Zepke and Leach, 2010; Shogren et al., 2018; Al-Jubari et al., 2019).

Deci and Ryan (2000, 2002) found that high autonomy and competency influence individuals’ actions according to their intrinsic motivation and enhance their interactions in interpersonal relationships through social relatedness (Jang et al., 2016; Trenshaw et al., 2016; Shogren et al., 2018). When the basic psychological needs of autonomy, competency, and social relatedness are met, individual adaptive outcomes will include well-being, vitality, high self-esteem, etc. (Jang et al., 2016; Aidinopoulou and Sampson, 2017; Sergis et al., 2018). If basic needs are not met, individuals will show characteristics of poor adaptation, such as anxiety and depression (Deci and Ryan, 2002). SDT integrates many social environmental factors and individual psychological variables that influence individuals’ learning, involvement, and well-being (Guay et al., 2008; Trenshaw et al., 2016). Hence, a plethora of empirical studies have been conducted based on SDT, and additional theoretical and academic contributions have been made in the education, health, and medical fields. For example, based on SDT, Vallerand et al. (1997) proposed a hierarchical model of intrinsic and extrinsic motivation that describes the motivation development process of individual action as social situation → psychological mediation → motivation mode → behavioral outcome. In the education field, student learning-related research about the mode has shown that self-determination positively influences the learning process (Shogren et al., 2018; Al-Jubari et al., 2019) and that behavioral and learning outcomes depend on student learning outcomes (Hummel and Randler, 2012). Based on the context above, SDT (Deci and Ryan, 2000) is adopted in this study to explore the psychological state of college students who grow up in the economically disadvantaged families from the need-based perspective (Shogren et al., 2018). This theory is used to explain the determined states of students in the learning process and examine whether students’ learning motivation is influenced by the difference in socioeconomic status, thus providing specific directions for the educators.

Learning outcomes are a key factor for the development inspection, which is the indicator by which learning outcomes of students are judged (Guay et al., 2008), and even the element of students’ learning evaluation and school’s satisfaction (Peterson and Einarson, 2001; Duque and Weeks, 2010; Sergis et al., 2018). Pike et al. (2011, 2012) pointed out that learning is a process that arises from actions taken based on experience. Thus, a strong social influence is generated by means of course participation and interaction with teachers or classmates, and students’ performance can be used as an evaluation indicator after their participation in learning activities. In analyzing how the expenditure of universities and the degree of student engagement influence students’ academic performance (Trenshaw et al., 2016; Sergis et al., 2018), Pike et al. (2011) presented two variables to measure student learning outcomes: cognitive gains and non-cognitive gains. Cognitive gains come from students’ experiences at the university and are conducive to general knowledge and skills, problem solving, information application, critical thinking, etc. Non-cognitive gains arise from students’ self-understanding, value judgments, cooperation with others, public participation, etc. Pike et al.’s (2011) measurement method assessed the academic performance of undergraduates via learning outcomes measured in terms of cognitive and non-cognitive gains.

### Learning Motivation

Learning motivation refers to students’ willingness to learn and engagement in the course, which can influence the decisive direction and emphasis in the learning process. Within the learning process, any degree of endeavor at any stage can be regarded as one learning activity. Since the prerequisite knowledge reserve of self-cognition of students in the learning process can influence their academic performance and predict changes in learning engagement (Cole et al., 2004; Jurik et al., 2014; Trenshaw et al., 2016), previous research on learning motivation has focused on the psychological cognition level (Chen et al., 2009). With regard to learning motivation, students with high motivation (high autonomy and low control) present better cognitive outcomes and higher learning achievement compared to students with low motivation (low autonomy and high control) (Vansteenkiste et al., 2009; Jurik et al., 2014).

SDT is an important psychological cognitive theory used to explain the students’ learning motivation and learning achievement (Trenshaw et al., 2016). Based on SDT, learning motivation derives from the demand for autonomous action and feelings of controllable self-action, and emphasizes the importance of intrinsic motivation (Deci and Ryan, 1985,2008,2000; Deci and Moller, 2005; Shogren et al., 2018; Al-Jubari et al., 2019). It can be divided into academic identity and role identity.

Students’ understanding and academic interests and abilities are considered their self-identity, also referred to as their individual academic identity (Deci and Ryan, 2000; Anctil et al., 2008). Research in the Western context has revealed that the academic autonomy and competency of students are important components of academic identity and that the corresponding academic identity, learning motivation, and engagement degree are associated with academic achievement (Boyd et al., 2003; Lounsbury et al., 2005).

### Relationship Between Learning Motivation and Learning Outcomes

Motivation is a necessary factor for individual learning engagement. If individuals lack the will to participate in the learning processes, they will not generate good learning outcomes (Tella, 2007). Scholars have not reached consensus in terms of how motivation promotes more proactive learning among students (Zepke and Leach, 2010; Trenshaw et al., 2016), however, Yorke and Knight (2004) suggested that self-belief is the key to motivation. That is, students high in self-belief are more driven to master concepts in order to achieve significant and efficient learning; this self-belief depends on their internal desire to acquire knowledge and gain a wide variety of skills (Campbell and Cabrera, 2014).

From the psychology perspective, students’ learning behaviors are based on their political beliefs, the society in which they live, and their academic motivation (Shogren et al., 2018). In relation to students’ learning behaviors, having academic motivation means that students have higher achievement goals (Tella, 2007); in this study, motivation corresponds to their role identity and academic identity. In other words, role identity and academic identity are conducive to motivating and maintaining the learning interests of students and can promote students’ deep thinking about the essence and implications of knowledge (Shogren et al., 2018). Yorke and Knight (2004) indicated that students with self-theories devote themselves to learning, which influences their learning motivation, ability, and engagement, and even their likelihood of goal achievement. When a goal is not feasible or cannot be realized, learning motivation and learning participation decrease. Llorens et al. (2007) planned a set of individual resource–efficiency–engagement, and found that students have higher self-efficacy and are more willing to devote themselves to learning when they believe they have individual resources and can complete a task (Trenshaw et al., 2016). However, several previous studies indicated that the low self-esteem and lack of autonomy and control may happen to economically disadvantaged college students because of their long-term weak position, so they tend to suffer more psychological predicaments such as aimlessness, learning disability, and hopelessness in the face of learning courses compared to their peers (Miller and Rottinghaus, 2014; Shogren et al., 2018). It can thus be assumed that students with different socioeconomic status select different learning activities due to their inconsistent learning goals and different understandings of the importance of learning, which leads to varied learning outcomes (Shogren et al., 2014; Shogren and Shaw, 2017; Shogren et al., 2018).

### Learning Mode

In previous studies about student learning, teachers’ questioning and feedback have been considered important teaching components and found to have profound implications for students’ learning process (Erdogan and Campbell, 2008; Voerman et al., 2012). Learning activities play an important role in students’ individual academic achievement (Perry et al., 2006; Pike et al., 2012). Hence, promoting learning effectiveness in students relies on building an environment that fosters student participation and engagement in learning activities, instead of enhancing students’ learning engagement via traditional teaching guidance.

In this study, the components of learning engagement include students’ energy, involvement, professional efficacy, and absorption (Schaufeli et al., 2002; Al-Jubari et al., 2019). Schaufeli and Salanova (2007) found that students with higher energy, greater mental resilience, and greater persistence in adverse learning environments can complete their schoolwork; realize the importance, foster enthusiasm for, derive inspiration from, and appreciate the challenge of learning via participation in activities; and better concentrate on individual study. Hence, teachers should change from teacher-directed methods to student-centered activities in order to enhance students’ learning and engagement, and guide students toward deeper understanding so that they can apply their knowledge in different situations (Lave and Wenger, 1991; Tagg, 2003; Aidinopoulou and Sampson, 2017).

With regard to the connotations and classification of students’ approaches to learning, scholars’ opinions vary (Duff et al., 2004). Nevertheless, results of extant research have indicated that deep learning is an important variable in predicting learning performance, as well as making students concentrate on essential content and fundamental implications, integration connection and metacognition. Students who only engage in surface learning adopt rote learning methods or only learn the knowledge needed for examinations and tests, instead of comprehensively understanding course content (Duff et al., 2004; Campbell and Cabrera, 2014). However, the predictive model of students’ academic performance has not been probed as deeply in classification-based research; that is, the deep learning approach is superior to the surface learning approach and may reduce the integrity of theoretical framework.

In addition, most scholars have emphasized that greater conversion and cooperative relationships between higher education and industry should be developed and that higher education institutions should make every effort to enable students to obtain relevant knowledge and skills through learning before working (Corbett, 2005; Philip et al., 2008). Such knowledge and skills can be divided into two categories (Hmelo-Silver et al., 2007). The first category is the practice ability. This entails sensitivity and imaginative thinking and emphasizes the soft skills of organization, communication, environment adaptation, and opportunity mastery (Corbett, 2005; Li et al., 2007). The second category is academic abilities. This entails logical thinking and emphasizes the deductive process of argumentation, induction, and theoretical innovation. In this study, the learning mode is divided into explorative learning and exploitative learning, which occur on the axis of theory and practice.

### Relationship Between Learning Mode and Learning Outcomes

**FIGURE 1:**
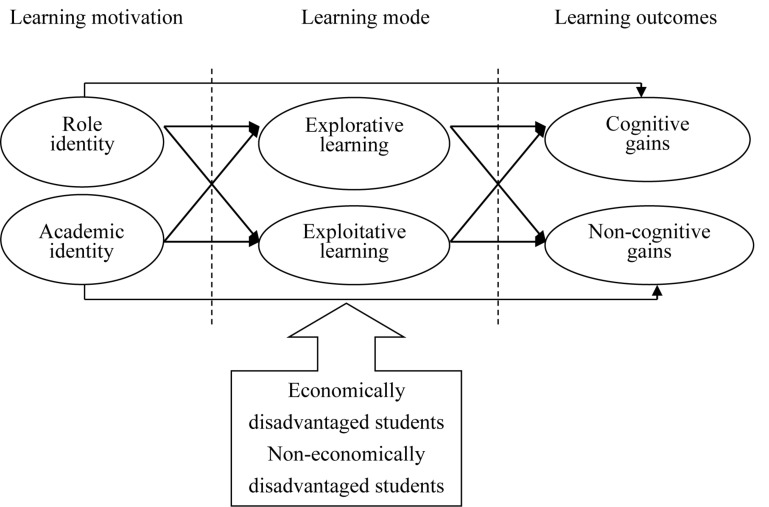
Research framework.

According to definitions given by scholars, explorative learning means that students are occupied in inquiries and tests of specialized knowledge theory via participation in the survey (Hmelo-Silver et al., 2007). Through such learning, they acquire reasoning skills in specialized subjects and analysis skills that can be applied in practice (Philip et al., 2008). Exploitative learning refers to a learning mode that occurs in the real world. In other words, knowledge is internalized by means of experience conversion, and is extracted, experienced, or inherited routinely to solve problems with higher efficiency or using more efficient methods (Corbett, 2005; Hmelo-Silver et al., 2007; Li et al., 2007). In this study, both explorative and exploitative learning are considered to generate a positive impact on student learning outcomes and are included in both the learning mode of students and course teaching (Hmelo-Silver et al., 2007). Hence, they are only different in investment proportion and priority in terms of the definition essence or operation. To sum up, in this study, the learning mode of students is classified as the basic educational situation based on theoretical principles and practical application, and the relationship between the learning method and learning outcomes is discussed, as showed in Figure 1. In this way, we contribute to the understanding of how to create the most suitable course plans and activities at universities. For this purpose, the following research questions are posed:

1. What are the differences between economically disadvantaged students and non-economically disadvantaged students regarding learning motivation, learning mode, and learning outcomes?
2. What mediating role do explorative learning and exploitative learning play in the model?
3. What are the differences between economically disadvantaged students and non-economically disadvantaged students regarding the various dimensions of the research framework?

## Research Method

### Sampling

The research sample in this study comprised undergraduates. Purposive sampling was adopted, since there are many mathematics departments in universities, and different universities have different theories on school management and different teaching characteristics. To understand whether the subject attributes would influence the research results, the different research variables between students majoring in natural sciences and those majoring in social sciences were verified. The results indicated that subject did not significantly impact the research variables, so it did not need to be included as an independent variable in subsequent analyses. Besides, the definition of “economically disadvantaged” varies in different countries. It is subject to the definition given in Taiwan, because this study takes undergraduates in Taiwan as the research object. The classification criteria of economically disadvantaged students are subject to the low-income household defined by the Ministry of Health and Welfare in Taiwan. The low-income household means the household with average monthly income per capita less than the absolute standard of living of 363.2 USD (10,869 TWD). Students beyond the scope of the low-income household will be classified into non-economically disadvantaged students.

Of the sampled representative universities in Taiwan, there are a total of seven national universities and nine private universities. To confirm whether there were differences in the responses between national and private universities, the present study conducted a non-response deviation test. The results of independent sample *t-*test show that there were no significant differences in the basic data of the main aspects, which means that there were no significant differences among the sample data of these universities. A questionnaire was issued in October and November 2018, and a total of 830 were returned; this included 13 invalid questionnaires, leaving 817 valid responses, as Table 1 showed. The economically disadvantaged students in the senior level are less than that in the sophomore and junior levels. A chi-square test was used to verify whether the samples of seniors versus sophomores and juniors differed significantly in terms of research dimensions. The results indicated that the two groups do not significantly differ, so it was deemed appropriate to merge the samples from different grades.

**TABLE 1:** Descriptive statistics by socioeconomic status and measurement scales.

Characteristic	Scale	Economically disadvantaged students	Non-economically disadvantaged students
Gender	Male	189 (52.5%)	242 (53.0%)
	Female	171 (47.5%)	215 (47.0%)
Part-time job	Yes	180 (50.0%)	147 (67.8%)
	No	180 (50.0%)	310 (32.2%)
Scholarship	Yes	168 (46.7%)	58 (12.7%)
	No	192 (53.3%)	399 (87.3%)
First-generation college student	Yes	262 (72.8%)	214 (46.8%)
	No	98 (27.2%)	243 (53.2%)
Majors	Social science	190 (52.8%)	248 (54.3%)
	Natural science	170 (47.2%)	209 (45.7%)
Dedication to class preparation	Yes	131 (36.4%)	308 (67.4%)
	No	229 (63.6%)	140 (32.6%)
Weekly study hours spent on	Less than 5	222 (61.7%)	141 (30.9%)
major courses	5 to less than 10	91 (25.3%)	124 (27.1%)
	10 to less than 15	25 (6.9%)	112 (24.5%)
	15 to less than 20	4 (1.1%)	49 (10.7%)
	More than 20	18 (5.0%)	31 (6.8%)
Active participation in class	Seven-point Likert scale from “Not at All” to “Very Active”	Mean = 4.358 *SD* = 1.421	Mean = 4.869 *SD* = 1.244
Involvement with teacher	Seven-point Likert scale from “Very Dissatisfied” to “Very Satisfied”	Mean = 4.628 *SD* = 1.338	Mean = 5.287 *SD* = 1.017
Teaching quality of teacher	Seven-point Likert scale from “Very Dissatisfied” to “Very Satisfied”	Mean = 4.806 *SD* = 1.331	Mean = 5.667 *SD* = 0.880

### Measurement of Variables

Academic identity refers to students’ understanding of their own schoolwork and to their capacity performance and is set by means of achievement goals to assess students’ willingness to participate in course learning and the learning serviceability. The academic identity scale in this study adopts four items used by Nasir et al. (2009). Students were required to indicate the extent to which they felt engaged in their major, course, schoolwork, etc.

Role identity refers to the level of identity constructed after the role cognitive development of students is influenced by the society environment. In this study, five items developed by White et al. (2008) were adopted to measure students’ participation in academic courses, and their belief level of role identity upon achieving an academic goal.

Since universities seek to promote the proactive learning of students via various teaching methods to enable students to obtain better learning outcomes, learning approach is divided into explorative learning and exploitative learning, as per previous literature in this field. For explorative learning, the explorative learning scale proposed by Philip et al. (2008) was adopted. The original scale was designed for students of a department of medicine; thus, the 10 items of the original scale were integrated into eight items in this study in order to enhance their generality. For exploitative learning, the exploitative learning scale proposed by Li et al. (2007) was adopted; this comprises 10 items in total, including aspects related to career preparation, time management, individual engagement, and satisfaction.

Student learning outcomes can be divided into cognitive gains and non-cognitive gains; for this, the scale proposed by Pike et al. (2011) was adopted. The cognitive gains scale contains nine items and require the students to point out their degree of progress in the process of experience learning at universities; the non-cognitive gains scale contains seven items. The variables for student learning outcomes were measured using a scale developed by Pike et al. This was based on characteristics of undergraduates in Europe and the United States, and its credibility and validity have been verified. Thus, the scale was considered suitable for expansion to the context of Asia; this will also verify the generalizability of the scale and improve its theoretical value for measuring learning outcomes. All of the above items used a seven-point Likert scale (1 = strongly disagree, 7 = strongly disagree).

A CFA measurement mode matching degree pointer roughly meets the recommended standards (chi-square/df = 2.82; GFI = 0.91 > 0.90; AGFI = 0.88 > 0.80; CFI = 0.96 > 0.90; RMR = 0.07 < 0.08; RMSEA = 0.05 < 0.08). The AVE dimensions are between 0.44 and 0.66, and the CR values are between 0.71 and 0.94. The matching degree of all variables is good. Thus, the research variables have good reliability and convergent validity, as shown in Table 2.

**TABLE 2:** Reliability and validity of the variables used in this study.

	1	2	3	4	5	6
Role identity	*(0.67)*					
Academic identity	0.45**	*(0.70)*				
Explorative learning	0.46**	0.41**	*(0.81)*			
Exploitative learning	0.45**	0.44**	0.81**	*(0.81)*		
Cognitive gains	0.37**	0.44**	0.72**	0.74**	*(0.66)*	
Non-cognitive gains	0.33**	0.39**	0.64**	0.66**	0.71**	*(0.69)*
Mean	5.22	5.21	5.04	4.89	4.96	5.08
*SD*	0.84	1.07	0.93	0.92	0.85	0.92
α	0.64	0.79	0.94	0.93	0.87	0.85
AVE	0.45	0.49	0.65	0.66	0.44	0.48
CR	0.71	0.78	0.94	0.89	0.87	0.85

*Values along the diagonal are the square root of the AVE for each dimension.* **p < 0.001. Numbers in italic denote the square root of AVE.

## Analysis and Results

### Difference Analysis Between Economically Disadvantaged and Non-economically Disadvantaged Students

A difference analysis was conducted for economically disadvantaged versus non-economically disadvantaged students, and all data were analyzed using SPSS 22.0. An independent samples *t-*test was adopted to test for differences between the two groups of students with regard to role identity, academic identity, explorative learning, exploitative learning, cognitive gains, and non-cognitive gains. The results are as follows: role identity (*t* = –8.02; *p* < 0.001), academic identity (*t* = –4.17; *p* < 0.001), explorative learning (*t* = –6.68; *p* < 0.001), exploitative learning (*t* = –7.94; *p* < 0.001), cognitive gains (*t* = –5.24; *p* < 0.001), and non-cognitive gains (*t* = –4.30; *p* < 0.001). The degree of cognition of non-economically disadvantaged students was thus significantly higher than that of economically disadvantaged students, indicating that students’ economic level influences their cognition.

### Analysis of the Mediating Effect Between Explorative Learning and Exploitative Learning

**FIGURE 2:**
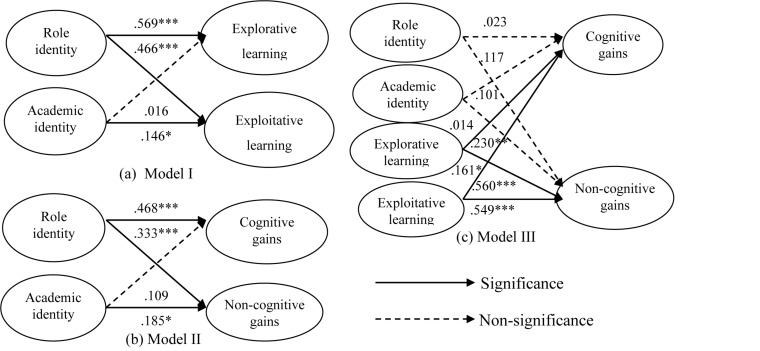
Verification of mediating effects. *p < 0.05, **p < 0.01, ***p < 0.001.

With respect to the learning process of students in higher education, learning motivation and learning outcomes must be included in the model to reveal the most appropriate learning method. Hence, in order to understand the mediating role between explorative learning and exploitative learning, mediating model verification was conducted using SEM as per the method proposed by Baron and Kenny (1986). This analysis is presented according to three competing models. As shown in Figure 2, all routes in Model I are significantly influential, except for explorative learning in relation to academic identity, which is not statistically significant. In Model II, role identity and academic identity generate a significant influence on both cognitive and non-cognitive gains [thus meeting condition (II)], but all paths are not significant [thus meeting condition (IV)] after explorative learning and exploitative learning are added to Model III. The above four groups of conditions show that exploitative learning has a full mediating effect on role identity and learning outcomes (cognitive gains and non-cognitive gains), as does exploitative learning on academic identity and non-cognitive gains. Based on the coefficient value between Model I and Model II, role identity has a more obvious influence on the mediator and dependent variables than does academic identity. This indicates that students feel they can obtain a sense of identity and meet the expectations of society and family as long as they play their expected role and meet their obligations, and will try their best to reach their goals even if they are studying an unfamiliar major.

The mediating effect verifies that exploitative learning plays an intermediary role between learning motivation and learning outcomes. The results indicate that students’ high learning motivation may not be directly reflected in their learning outcomes, and that learning outcomes can be promoted only via actual application of learning. In addition, role identity and academic identity do not have a significant influence on cognitive and non-cognitive gains (Model II in Figure 2) in the model. The research results also verify the key mediator of learning mode. Hence, the path relationship between learning motivation and learning outcomes is not discussed in the subsequent comparison of path relationship.

### Comparison of the Path Relationship Between Economically Disadvantaged and Non-economically Disadvantaged Students

The path relationship among all dimensions was estimated via multi-group SEM, wherein the path value adopted standardized coefficients to verify the eight paths of the research model. The path analysis coefficients of the structural model for economically disadvantaged versus non-economically disadvantaged student samples are shown in Table 3. In the path relationship between economically disadvantaged and non-economically disadvantaged students, it is found that role identity has a positive, significant effect on explorative learning and exploitative learning; explorative learning and exploitative learning positively influence cognitive gains; and exploitative learning has a significant positive impact on non-cognitive gains. However, no significant effect is found between, for example, explorative learning → non-cognitive gains of economically disadvantaged students; or the academic identity → explorative learning/exploitative learning, or explorative learning→cognitive gains/non-cognitive gains, of non-economically disadvantaged students.

**TABLE 3:** Path relationship comparison between economically disadvantaged and non-economically disadvantaged students.

Path relationship	Path coefficient value		*t-*value of path relationship comparison
	
	Economically disadvantaged students	Non-economically disadvantaged students	
Role identity → explorative learning	0.324**	0.664***	−37.82***
Role identity → exploitative learning	0.225**	0.549***	−36.95***
Academic identity → explorative learning	0.188**	–0.053	32.39***
Academic identity → exploitative learning	0.340**	0.119	29.01***
Explorative learning → cognitive gains	0.184**	–0.019	36.51***
Explorative learning → non-cognitive gains	0.024	0.007	2.42**
Exploitative learning → cognitive gains	0.680***	0.891***	−34.59**
Exploitative learning → non-cognitive gains	0.747***	0.818***	−9.42***

**p < 0.01, ***p < 0.001.

The sample data were grouped according to the economic attributes of the students’ families. In order to further test whether the model path relationship between economically disadvantaged students and non-economically disadvantaged student samples is significantly different, an approach proposed by Tsang (2002) was adopted. This made it possible to identify the significance of the path relationship among the models via a pooled *t-*test. The operation process of the *t* value is shown in the following equations. As shown in Table 3, the relationship comparison of all paths reveals statistical significance, except for role identity → explorative learning (*t* = –37.82, *p* < 0.001), role identity → exploitative learning (*t* = –36.95, *p* < 0.001), exploitative learning → cognitive gains (*t* = –34.59, *p* < 0.001), and exploitative learning → non-cognitive gains (*t* = –9.42, *p* < 0.001). This indicates that non-economically disadvantaged students have significantly higher self-expectations with regard to role obligation, and demand for exploitative learning mode. As a result, their path relationships are significantly higher than those of economically disadvantaged students. Nevertheless, in the sample of economically disadvantaged students, their paths with positive statistical significance are more than those of non-economically disadvantaged students. The results of the pooled *t-*test show that the path relationship between the learning identity and learning mode of economically disadvantaged students is significantly higher than that of non-economically disadvantaged students, especially for explorative learning → cognitive gains (*t* = 35.51, *p* < 0.001), which reveals the greatest difference. This means that economically disadvantaged students have comparatively higher learning growth in the explorative learning mode. Hence, with respect to learning motivation, learning mode, and learning outcomes, non-economically disadvantaged students do not exhibit superior results in all dimensions. Thus, economically disadvantaged students can be stimulated and guided to obtain learning outcomes that are superior to those expected by strengthening certain learning motivation factors.

$$S_{p\,o\,o\,l\,e\,d}=\sqrt{{\frac{N_{1}-1}{N_{1}+N_{2}-2}}^{*}\,S\,E_{1}^{2}+{\frac{N_{1}-1}{N_{1}+N_{2}-2}}^{*}\,S\,E_{2}^{2}}\;$$

$$t=\frac{P\,C_{1}-P\,C_{2}}{S_{p\,o\,o\,l\,e\,d}^{*}\,\sqrt{\frac{1}{N_{1}}+\frac{1}{N_{2}}}}$$

*S*_*pooled*_: pooled estimated value of standard deviation; *SEi*: sample standard error; *PC*_*i*_: path coefficient value; *N*_*i*_: number of samples; *t*: coefficient value of *t*-test.

## Conclusion

### Discussion

Economically disadvantaged students are restricted in accessing education resources and opportunities due to the adverse conditions they face in terms of socioeconomic status, family, culture, etc. (Baker and Logan, 2006). As a result, they often feel incapable of learning, lack confidence, and experience heavy pressure, while having difficulty in terms of schoolwork, skill cultivation, and learning performance. Hence, it is worth further discussion as to whether economically disadvantaged students can enhance their development and meet their full potential in terms of academic achievement and employment competitiveness. There are different cognition in analysis of learning process between economically disadvantaged and non-economically disadvantaged students, and differences in the two groups’ learning motivation, learning methods, and learning outcomes were verified via an independent samples *t-*test. The results show that non-economically disadvantaged students have a higher cognition degree with respect to learning variables compared to economically disadvantaged students, which indicates that the former group’s lack of resources influences their learning outcomes. In other words, students with fewer resources have to earn their tuition or living expenses, for example, through part-time jobs in order to meet their daily needs. As shown in the basic sample data of this study, the percentage of economically disadvantaged students taking part-time jobs is 50%, which is higher than the 32.2% of non-economically disadvantaged students with part-time jobs. The off-campus working environment is complex, and problems often arise in terms of working safety and time distribution. In addition, off-campus part-time jobs indirectly reduce students’ engagement in their academic course, which may lead to a higher drop-out rate and lower graduation rate among this group (Kassier and Veldman, 2013). The vicious circle generated by the above phenomenon is called the Matthew effect.

In the different samples of economically disadvantaged and non-economically disadvantaged students, the results pertaining to the path relationship of the model in this study indicate that role identity has a positive and significant influence on both explorative learning and exploitative learning and show that students’ engagement in various learning activities increases when they are fully aware of their roles and obligations as students. As also found by Petersen et al. (2009) and Jang et al. (2016), this study emphasizes that self-determination, motivation, self-esteem, a lack of perceived pressure, and other important individual internal factors contribute to students’ learning. This conclusion can also be used to measure the key adjustment direction at universities to replace extrinsic reward with intrinsic motivation.

Chinese college students may have a stronger drive toward academic achievement due to a sense of obligation and social pressure. However, in this study, the academic identity of non-economically disadvantaged students was found not to have a significant effect on explorative learning and exploitative learning. This finding is not fully consistent with the Western achievement motivation theory, but it conforms to the cultural perspective emphasized by Chen et al. (2009). In other words, assessments of learning motivation as considered in relation to psychological theory must take into account cross-cultural factors and specialty (Chang et al., 2011). Chinese students often face a certain degree of role obligation when pursuing their studies. Therefore, the degree of autonomy of Chinese students may be more restricted by social desirability compared with that of Western students, although Chinese students are likely to develop independent interests or a sense of competence in relation to achieving academic goals. In addition, Chinese students may regard successful academic performance as a responsibility in order to meet parents’ expectations when facing the high role obligations formed by the longitudinal goal. They may persuade themselves to strive to meet such obligations even if they have low intrinsic motivation to do so, or are even incapable of doing so. Thus, students may face an “underachiever’s dilemma.” Unsuccessful academic experience may change an interest in learning into a sense of responsibility or obligation, which may lead to undesirable psychological traits or behavior in the long run and reduce students’ engagement in explorative and exploitative learning.

The results of the pooled *t-*test indicate that economically disadvantaged students have lower relationship intensity between role identity and explorative and exploitative learning compared to non-economically disadvantaged students. This result is in line with the achievement motivation proposed by Chen et al. (2009); that is, the expectation and the social value of members of a group will have different influences on the development of their achievement goal, thus deriving important implications and results for their learning engagement (Guay et al., 2008). In other words, non-economically disadvantaged students are highly affected by social desirability and role obligation from family or parents, so may more easily perceive the role they are supposed to play and more intensely commit to learning activities compared to economically disadvantaged students.

Few achievement motivation theories probe students’ self-competency, expectations of success, and subjective value of goals via economic factors. However, non-economically disadvantaged students have greater social support and education resources, as well as higher self-expectations and more self-set goals. As a result, they may face a greater sense of loss, anxiety, and dampened enthusiasm and confidence when facing setbacks, which will influence their learning performance and selected learning method.

This study also considered the relationship between students’ learning mode (explorative learning versus exploitative learning) and learning outcomes (cognitive gains versus non-cognitive gains), and the method that contributes to the improvement of learning outcomes of economically disadvantaged versus non-economically disadvantaged students upon application of the explorative learning and exploitative learning mode. The results are divided into two parts according to the variables considered. First, both explorative learning and exploitative learning have positive and statistically significant influences on cognitive gains. This means that the two learning methods play an important role in knowledge acquisition and are involved in the process of controlling feedback mechanisms and constantly amending errors. Furthermore, they can serve to upgrade students’ individual memory via conscious or unconscious learning, promote the application of explorative and exploitative learning, and strengthen students’ efficiency via cognitive gains and non-cognitive gains by helping them to obtain specialized knowledge, make theoretical deductions, engage in deep discussion of theory, and cultivate time and pressure management and other skills (Hmelo-Silver et al., 2007; Li et al., 2007). Second, with respect to non-cognitive gains, both economically disadvantaged and non-economically disadvantaged students can take part in exploitative learning activities. Teachers are responsible not only for imparting academic knowledge to students in terms of affective development, moral education, and value building, but also for guiding students to cultivate a positive attitude, social soft skills, and morality when interacting with other people and dealing with matters outside of the classroom (Zepke and Leach, 2010; Pike et al., 2011, 2012).

Nevertheless, in this study, the relationship between explorative learning and non-cognitive gains of economically disadvantaged and non-economically disadvantaged students was found to be statistically insignificant, probably because the non-professional characteristics of students, such as individual morality and values, are greatly influenced by families or peers. Such attitudes and values are difficult to change even when they gain more specialized knowledge or problem-solving strategies. Hence, teachers and schools must seek to increase students’ knowledge and strengthen their ability to complete schoolwork or extracurricular problems cost effectively in order to reduce the possibility of problem-solving error, and the evaluation risk of various decisions. As a result, students will make better cognitive and non-cognitive gains.

The mediating effect of explorative learning and exploitative learning was probed in this study via the test items proposed by Baron and Kenny (1986). The results show that exploitative learning has a complete mediating effect on the learning motivation and learning outcomes of students. In other words, learning motivation may not significantly promote student learning outcomes, and learning outcomes can be achieved by adopting an appropriate learning method or sufficiently engaging in learning activities (Petersen et al., 2009).

Since exploitative learning plays an important coordinating and shifting role in learning motivation and learning outcomes, college teachers, in addition to imparting theoretical knowledge on their subject, should attach importance to practical application of knowledge. Theoretical knowledge is built on the interpretation of phenomena, constant argument, and the propositions and opinions of scholars, however, theory must be updated constantly, or new theories must be introduced to explain phenomena as people continue to be influenced by environmental variables. Thus, teaching must be continuously innovated as well (Oleson and Hora, 2014). Students must not only learn theoretical knowledge, but also think about the origin of problems and their implications, define problems or phenomena based on theory, and solve problems by applying knowledge gained.

### Practical Implications

Based on the research results, several practical suggestions are suggested in order to strengthen the learning motivations and learning methods of both economically disadvantaged and non-economically disadvantaged students.

The learning process of economically disadvantaged students was explored on the basis of psychological motivational factors. Although motivation is an important factor influencing learning methods, and schools must make every effort to promote students’ role identity and academic identity, these two motivation factors are sufficient, but not necessary, conditions to stimulate the learning of economically disadvantaged students. Universities are suggested to also provide external assistance or economic support – that is, psychological motivations for learning can be triggered on the basis of economic conditions. Universities can provide stable part-time jobs or internship opportunities on campus so that students, in addition to earning money to cover living expenses, can stay in school to gain subject-related practical experience or take part in campus service learning, develop learning interest, and obtain professional knowledge and skills. Moreover, the academic follow-up and learning engagement of students will not be undermined by their having a job in an unsafe workplace, or having to take overtime.

In addition, teachers’ expectations and school support considerably influence the role identity formation of economically disadvantaged students. Thus, teachers can provide recognition and guidance to enhance the self-learning of economically disadvantaged students by designing a positive course based on peer learning, and can evaluate students’ role cognition via multiple assessment methods (White et al., 2008). In other words, course participation and engagement, rather than academic scores, should be emphasized.

Generally speaking, universities still attach insufficient importance to liberal education, and course planning typically fails to include teaching activities and content specifically related to social adaptability, soft skills for employment, ethical values, etc. This indirectly reduces opportunities for non-economically disadvantaged students to cultivate ethics, values, and self-belief. Hence, universities, in addition to strengthening the teaching of professional knowledge, should cultivate students’ social competence and provide career coaching, set up career planning courses, guide students in their self-understanding and exploration, and enhance the positive learning experience by imparting ethical values and encouraging social adaptation via club activities, volunteer service, and other informal opportunities.

In addition, non-economically disadvantaged students were found to have a weaker path relationship between explorative learning and learning outcomes compared to economically disadvantaged students. The difference analysis showed that the cognition degree of explorative learning (*M* = 5.23) was higher than that of exploitative learning (*M* = 5.11); however, continuous and abundant professional theoretical learning may cause lower effect in learning curve. As a result, non-economically disadvantaged students have fewer comparative advantages with regard to learning outcomes compared to economically disadvantaged students. Thus, universities are suggested to utilize the online environment to present learning data via the Web, and to provide multiple and flexible learning paths and opportunities in order to attract students’ attention. In addition, universities can utilize information technology to standardize the work flow through effective data sharing platforms or electronic databases. All of these approaches could contribute to simplifying complex knowledge, as well as enabling students to share individual learning experiences via these platforms (Philip et al., 2008). Thus, non-economically disadvantaged students can apply the explorative and exploitative learning mode, and integrate the various knowledge and abilities acquired via the two methods, to enhance their cognitive and non-cognitive gains.

Application-oriented course designs focus on practical teaching models. This entails the application of theoretical models and content through real situational exercises or events. Hence, college teachers are suggested to guide students to participate in learning via case orientation, peer interaction, or practical tasks as part of an applied course design, and replace recitation with application, supplemented by relevant knowledge and description of the subject that includes examples or discussion. Only by doing so can students obtain better learning outcomes. In addition, teachers are encouraged to innovate their teaching methods, create explorative and exploitative learning modes via appropriate courses, and improve the linkage between specialized knowledge and practical application through activities and job placements.

This study assessed role identity and academic identity based on SDT in combination with the achievement of goals. Economically disadvantaged students were found to have a positive effect on learning mode under the influence of role identity and academic identity. This indicates that students still look forward to learning, but spend more time earning money to cover living expenses due to financial difficulties faced. Thus, universities are suggested to set up social ethics-related courses to augment general education courses, improve students’ psychological cognition by helping them to understand their role obligations, and establish a strong learning culture. As a result, economically disadvantaged students can change their role cognition and benefit from enhanced learning motivations and outcomes.

### Research Limitations and Future Research Directions

The research results contribute to the literature on economically disadvantaged students, SDT, and student learning outcomes; nevertheless, some limitations still exist and represent further research directions. First, motivation theory has obtained considerable status in the psychological field, but only a few studies have considered the relationship between learning motivations and learning outcomes of undergraduate students in higher education. Although the learning motivation dimension (role identity and academic identity) was constructed with reference to SDT in this study, and important learning theories can be derived from the research results, other motivation theories, such as attribution theory, self-efficacy theory, and hierarchy needs theory, still apply to explain how to trigger learning in economically disadvantaged students. Thus, it is suggested that future research can utilize different theoretical models in order to identify relevant motivation dimensions influencing students’ learning outcomes. Second, this study required students to self-report details on their academic achievement as the academic performance indicator, mainly because actual academic achievement data are confidential and not easily obtained. However, errors may exist in the students’ self-statement of their academic performance. The link between learning motivation and academic performance may be better understood if students’ actual academic performance is assessed, with due consideration for research ethics. Besides, this study suggests future researchers to include interview contents and economically disadvantaged students’ observations of learning status in their studies to support the researching results and make a comprehensive judgment. Third, due to restrictions of time and space, only 16 universities were sampled in this study, with 817 valid questionnaires in total. The research objects were divided into economically disadvantaged and non-economically disadvantaged students. Future research could explore and compare other groups, in addition to expanding the quantity of samples and improving the research representativeness, so as to provide additional insights relevant to higher education policy.

## Data Availability Statement

The raw data supporting the conclusions of this article will be made available by the authors, without undue reservation, to any qualified researcher.

## Ethics Statement

The studies involving human participants were reviewed and approved by Institutional Review Board, University of Taipei. The patients/participants provided their written informed consent to participate in this study.

## Author Contributions

HL and MP contributed to the ideas of educational research, collection of data, and empirical analysis. MP and MY contributed to the data analysis, design of research methods, and tables. C-CC participated in developing a research design, writing, and interpreting the analysis. All authors contributed to the literature review and conclusion.

## Conflict of Interest

The authors declare that the research was conducted in the absence of any commercial or financial relationships that could be construed as a potential conflict of interest.
